# The locus coeruleus, a blue spot for early diagnosis and prognosis of Alzheimer’s disease

**DOI:** 10.3389/fnagi.2025.1632236

**Published:** 2025-12-01

**Authors:** Fatemeh Farahani, Christelle Anaclet, Hossein Azizi, Heinrich S. Gompf

**Affiliations:** 1 Department of Pharmacology, Tulane University School of Medicine, New Orleans, LA, United States; 2 Department of Neurological Surgery, School of Medicine, University of California, Davis, Davis, CA, United States; 3 Department of Physiology, Faculty of Medical Sciences, Tarbiat Modares University, Tehran, Iran; 4 Institute for Brain and Cognition, Faculty of Medical Sciences, Tarbiat Modares University, Tehran, Iran

**Keywords:** locus coeruleus, vulnerability, neurodegenerative disease, noradrenaline, biomarker, Alzheimer’s disease

## Abstract

Intact functioning of the locus coeruleus-noradrenergic (LC-NA) system is pivotal in the control of numerous central processes, and damage to these systems leads to a wide range of nervous system disorders. The LC, as the main source of noradrenaline (NA) in the mammalian brain, was the first central nervous system (CNS) modulatory structure to be anatomically and biochemically characterized. LC-NA release exerts both excitatory and inhibitory effects on target areas. Over the past few decades, LC damage has been causatively identified as a common factor in CNS diseases, notably neurodegenerative diseases. Moreover, LC damage is the likely manifestation of common pathophysiological processes, thus elevating the LC as a diagnostic and therapeutic target, especially in Parkinson’s and Alzheimer’s diseases (PD & AD). This review also addresses why LC neurons, compared to other areas in particular, are highly vulnerable and sensitive to damage—such as specific anatomical features, tau phosphorylation, and high neuronal energy requirements—will be described in this review article. Finally, we explore whether these known LC vulnerabilities might be leveraged towards improved early diagnostic and prognostic biomarkers for AD.

## 1 Introduction

The activity of the brain’s neuromodulatory systems affect arousal, mood, attention, and motivation and consequently influence the behavior. The locus coeruleus (LC; “blue spot” in Latin) is the main hub of central noradrenaline (NA) which, as a key neuromodulator, affects forebrain-related cognitive functions and behaviors, including working memory, selective attention, decision-making, and adaptive behavior (Mather and Harley, 2016). Considering that several neurodegenerative and psychiatric disorders in humans are tightly related to disruption of NA signaling, it exemplifies the clinical importance of the LC-NA system (Sara, 2009; Feinstein et al., 2016). Other recent review articles explore technological advances that either allow a deeper functional understanding of the LC (Poe et al., 2020), or advanced imaging of the LC and other neuromodulatory areas (Engels-Dominguez et al., 2023; Galgani et al., 2020). Here, we provide updated references to both of these areas and focus on the very early stages of Alzheimer’s that impact the LC.

Discovery of the LC dates back nearly 240 years. The preeminent comparative anatomist and neuroanatomist of the 18th century, Félix Vicq d’Azyr, was the first to depict a dark blue-ish area at the base of the fourth ventricle in axial sections of the human brain drawn by Alexandre Briceau and published in 1786 [Planche XXIX, Figure III, area 12; (Vicq d’Azyr, 1786; Tubbs et al., 2011)]. In 1809, German anatomist Johann Christian Reil offered a descriptive location (“wo der vördere Schenkel mit der Area zusammenstößt”) of a darker tissue (“schimmert ein Strich von Schwarzer Substanz durch, die bloß mit dem Epithelium bedeck ist.”) (Reil, 1809). The translation offered in Chandler et al. (2019) is reminiscent of d’Azyr’s drawings: “In the angle at which the anterior shank [the *Pedunculus cerebellaris superior*] comes together with the adjoining area, a stripe of black substance shimmers through, only covered by the epithelium.” Writing in Latin in 1812 and using the alternate spelling “loculi caerulei” (Swanson, 2015), the brothers Joseph and Karl Wenzel described an area at the base of the fourth ventricle as ‘oblong, narrow bluish place [that] immediately strikes the eye at first sight’ (original Latin on p. 168: “angustus et caeruleus locus primo statim obtutu oculos ferit”) (Wenzel and Karl, 1812). In 1909, Jacobsohn described one main and two ancillary “nucleus pigmentosus pontis” but clearly identified the “triangular” main division in the corner of the fourth ventricle as “nucleus pigmentosus loci coerulei” worthy of particular attention due to its “brown[−ish] Pigment” (Jacobsohn, 1909). Additional details about the early history of the LC can be found in (Maeda, 2000). Importantly, the prominent dark, brown, or blue color, depending upon the preparation, is due to the accumulation of neuromelanin, formed through the oxidation and polymerization of catecholamines and their metabolites, principally dopamine and norepinephrine. It is this feature that attracted so many early neuroanatomists to the LC, which is hypothesized to be one of the reasons for its sensitivity to neurodegeneration (Sulzer et al., 2018), and potentially its use as an early biomarker of neurodegeneration in Alzheimer’s disease (AD).

### 1.1 Functional anatomy of the LC nucleus

The bilateral nucleus of the LC is located on the ventrolateral surface, as stated above ‘in the corner,’ of the fourth ventricle in the pons (Williams et al., 1984). Dimensions of this tiny nucleus in humans are estimated to be 12.0–17.0 mm, 2.5 mm, and 2.0 mm in length, width, and height, respectively (Fernandes et al., 2012). Neuronal nodes located in the brainstem containing NA are called “noradrenergic cell groups.” They include A1-A7, with the A6, locus coeruleus, being the largest among them (Dahlström and Fuxe, 1964). This tiny nucleus consists of compact cells, estimated to be around 45,000–50,000 in both hemispheres of the healthy adult human brain (Sharma et al., 2010), and although some variation has been noted, older individuals tend to have fewer LC neurons, which is more pronounced in those with dementia (German et al., 1988; Mouton et al., 1994; Kelly et al., 2017). The LC is composed of neurons that are mainly medium-sized cells with a fusiform and polar morphology, and smaller-sized neurons of various shapes (Chan-Palay and Asan, 1989; Patt and Gerhard, 1993). The medium sized neurons are projection neurons that suffer the most dendritic, axonal and cell body losses in neurodegenerative diseases (Williams et al., 1984; Chen et al., 2022). On a broader, “dendroarchitectonic” level, the LC is generally considered part of set of brainstem and pontine nuclei referred to as the “isodendritic core” contained within the reticular system as termed by Enrique Ramón-Moliner (Geser et al., 2022). Of the three neuronal types identified by Ramón-Moliner (allo- and idio-dendritic being the other two), the isodendritic neurons have the least dendritic complexity and thus could be hypothesized to be the most phylogenetically conserved form of neurons present in mammals (Ramon-Moliner and Nauta, 1966). Similar to other areas of the isodendritic core, the LC innervates throughout the neuraxis with volume transmission, and it is this combination of anatomical features that has been proposed to make this entire class of neurons particularly vulnerable to tauopathies (Gambardella et al., 2017; Lew et al., 2021) and AD pathogenesis (Giorgi et al., 2017; Theofilas et al., 2015). The LC itself is a mostly homogenous nucleus in which most neurons release NA (Szabadi, 2013; Taylor and Westlund, 2017), with simultaneous expression of a variety of neuropeptides, including neuropeptide Y, somatostatin, and cholecystokinin in a subset of neurons (Benarroch, 2018). Later studies on LC have asked how a small, homogeneous structure can respond to various sensory stimuli, modify neuronal actions in separate brain areas, and induce different behaviors. These studies have highlighted some heterogeneity within the LC-NA system (Schwarz and Luo, 2015; Chandler et al., 2014).

NA is one of the first neurotransmitters identified in the CNS, which was discovered and introduced by the Swedish physiologist Ulf von Euler in the 1940s (Von Euler, 1946). Apart from the studies that two Japanese researchers published during World War II, the first study about LC function was done by Russell in 1955 (Maeda, 2000). NA is released by LC neurons in two ways, synaptic and non-synaptic (volume) transmission. The latter involves the diffusion of NA into the extracellular space and the paracrine effects on neurons, glial cells, and capillaries (Waterhouse et al., 1977). The LC-NA system’s effects are mainly exerted by different types of NA receptors and their distribution in the LC target areas. Noradrenoceptors belong to three families of G protein-coupled receptors, including alpha-1, alpha-2, and beta, which operate with different mechanisms and each one has several subtypes (Benarroch, 2018). Various studies have reported multiple effects of NA on membrane potential, cell excitability, intracellular signaling cascades, and synaptic plasticity in target regions of the LC projections (Sara, 2009; Feinstein et al., 2016).

LC neurons, despite their small number, project throughout the neuraxis with the exception of the striatum, nucleus accumbens, substantia nigra, and globus pallidus. In addition, based on more recent studies, these neurons receive direct input from more than one hundred brain regions (Schwarz and Luo, 2015; Schwarz et al., 2015). For this reason, the LC plays a key role in regulating various functions such as the autonomic system, vigilance, sleep/wake cycle, sensory processing, attention, memory and learning, pain processing, and drug dependence (Poe et al., 2020; Szabadi, 2013; Suárez-Pereira et al., 2022; Alaee et al., 2022; Farahani et al., 2021; Farahani et al., 2023; Ahmadi-Soleimani et al., 2020; Samuels and Szabadi, 2008; Foote and Berridge, 2019; Aston-Jones and Waterhouse, 2016).

### 1.2 Physiology of the LC nucleus

LC neurons are electrically connected via gap junctions that respond to environmental stimuli (de Carvalho et al., 2014), and their electrophysiological activity is modulated by the synaptic release of various neurotransmitters. LC activity is sculpted by both subcortical inputs—for example, the orexinergic inputs from the lateral hypothalamus and corticotropin-releasing peptide from the central nuclei of the amygdala—as well as descending glutamatergic projections from the prefrontal and anterior cingulate cortices (Gompf et al., 2010). Furthermore, NA released from LC neurons provides feedback inhibition via alpha-2 autoreceptors, which are expressed in the cell bodies and dendrites (Benarroch, 2018; Benarroch, 2009).

In addition to the above-mentioned extent of LC projections, their distribution in the cortex is also heterogeneous. For example, in humans the most extensive innervations of the LC project to the somatosensory and motor cortices and associative regions including prefrontal and parietal cortices, and the temporal cortex, which is critically involved in memory formation (Lewis and Morrison, 1989; Gaspar et al., 1989). Using these connections, LC can contribute to alertness, response to stress, attention, and memory.

LC neurons fire action potentials in two distinct patterns, tonic and phasic. By switching between these two modes, different behavioral states are regulated. Basal tonic activity is characterized by continuous high-frequency (2–5 Hz) discharge in the awake and alert state. In quiet wakefulness, LC neurons fire regularly and at a low frequency of approximately 1 Hz, while they fire bursts in response to alerting stimuli. From drowsiness to slow-wave sleep, their firing frequency decreases, and LC neuronal activity is silent during REM sleep until the transition back to wakefulness. This ability to change firing patterns is crucial in maintaining the organism’s behavioral adaptation to environmental changes (Sara, 2009; Aston-Jones and Cohen, 2005).

During selective attention, the LC nucleus may temporarily stop its tonic firing and respond to the desired stimulus with phasic firing. This transient phasic firing pattern moderately elevates NA concentrations which in turn facilitates working memory formation mainly by acting on high affinity alpha-2 receptors. On the other hand, high concentrations of NA, resulting from augmentation of tonic firing, which is thought to occur during stress, activate medium-affinity adrenoceptors, alpha-1, and may cause defects in the function of the prefrontal cortex (Berridge and Waterhouse, 2003; Arnsten et al., 2012).

The role of the LC in the stress response is first manifested by the effect of NA on the beta receptors of the basolateral part of the amygdala. In addition, the inputs of the central nucleus of the amygdala to the LC are involved in increasing LC activity following stress. Indeed, laboratory evidence shows that the stress-induced activity of orexinergic neurons of the hypothalamus activates the orexin type 1 receptor in the LC. NA also contributes to the manifestations of autonomic system activity, such as heart rate and blood pressure. The LC is involved in the processing and modulation of pain through its connections with pain-processing centers such as the dorsal horn of the spinal cord, sensory nuclei of the trigeminal nerve, and the thalamus (Szabadi, 2013; Samuels and Szabadi, 2008; Barsegyan et al., 2014).

These afferent, efferent and intra-LC pathways may impact symptomatology throughout the course of AD. For example, the amygdala, another brain area displaying early susceptibility to AD pathology, influences network connectivity to various cortical regions, and also subcortical structures such as the LC, leading to neuropsychiatric symptoms throughout the course of AD (Stouffer et al., 2024). Numerous pharmacotherapies have been proposed for neuropsychiatric symptoms in AD (Pless et al., 2023). Amongst these symptoms, primarily apathy and anxiety in AD are likely to benefit most from noradrenergic treatment regimens (David et al., 2022; Liu et al., 2018; La et al., 2019); however, depression (Herrmann et al., 2004) and to some degree psychosis (Orlando et al., 2023) associated with AD have been shown to benefit from pharmacotherapies that target multiple monoaminergic systems, including noradrenergic receptors. For instance, tricyclic antidepressants that target norepinephrine and serotonin for depression, and atypical antipsychotics for psychosis, though the latter is highly controversial due to the negative side effects in older individuals. Since multiple noradrenergic target sites are coordinated by LC activity, LC degeneration is expected to affect larger-scale network reconfiguration, and thus functional connectivity studies might inform future drug intervention strategies. Although functional connectivity mapping of the LC has been hindered by its relatively small size (Serra et al., 2018), proxies to LC connectivity such as pupil diameter could be developed for AD research studies and patient diagnostics instead of LC fMRI to overcome the limitations of its small size (Wu et al., 2025). As discussed below, higher spatial resolution fMRI studies should begin to answer to which degree LC connectivity correlates with early disease biomarkers such as *APOE4* (Um et al., 2024) as well as symptomatic ramifications of AD, as has already been demonstrated for the role of the LC in depressive symptoms (Dai et al., 2023) and agitation (Liu et al., 2025) in AD, and in ageing more generally (Jacobs et al., 2018; Mijalkov et al., 2025; Vereb et al., 2023).

## 2 Vulnerability of LC neurons

Cell body abnormalities and loss in Parkinson’s disease (PD) were first noted in the 1950s, around the same time as comparable alterations were observed in postmortem aged and AD tissue (Clewett et al., 2016; Gesi et al., 2000). After that, LC degeneration and the subsequent reduction of NA in the CNS have been reported in a wide range of additional neurological disorders and neurodegenerative diseases, including anxiety, depression, sleep disorders, post-traumatic stress disorder, Huntington disease, and multiple sclerosis (MS), which is primarily a neuroinflammatory disorder rather than a classic neurodegenerative disease, as well as AD (Bielau et al., 2012; Chandley et al., 2013; George et al., 2013; Leiman and Hämmerer, 2024; Mann et al., 1982; Pietrzak et al., 2013; Polak et al., 2011; Rajda et al., 2006; Zhu et al., 2015; Zoukos et al., 1992; Zweig et al., 1992). A large part of the studies conducted on LC degeneration have been done in relation to AD. Histological evidence indicates that the number of LC neurons decreases by 60% in AD patients (Mann et al., 1982; Tomlinson et al., 1981; Perry et al., 1981) and up to 80% in PD patients (Zarow et al., 2003). It is important to note that the LC shares similar vulnerability to AD pathology and a number of other neurodegenerative diseases, including PD, and that other nodes of the isodendritic core share vulnerabilities that are seen in the LC. A number of review articles provide information about these issues that are outside of the scope of this review (Theofilas et al., 2015; Ehrenberg et al., 2023; Grinberg et al., 2011).

LC neurodegeneration is significantly correlated with the number of plaques, the duration and severity of dementia; it is substantially greater than other subcortical nuclei such as the substantia nigra, and is seen in earlier stages of the disease (Zarow et al., 2003; Bondareff et al., 1987; Grudzien et al., 2007). Concomitantly, the loss of LC via experimental lesions leads to greater amyloid deposits and increased neuroinflammation in LC projection areas (Heneka et al., 2006; Heneka et al., 2010). Therefore, both the onset and progression of AD pathogenesis are intertwined with LC degeneration (Beardmore et al., 2021). In this section we discuss the possible reasons for the vulnerability of these neurons. We later turn our attention to how these known vulnerabilities can serve as prognostic tools in the care and treatment of AD patients.

### 2.1 LC degeneration during aging

LC degeneration is a normal part of aging, though it is accelerated in AD. Progressively through the fourth decade of life, 25%–40% of pigmented LC neurons are lost, most prominently in the rostral part of this nucleus (Chan-Palay and Asan, 1989; Mann et al., 1982; Vijayashankar and Brody, 1979; Manaye et al., 1995), and this starts earlier in men compared with women (Wree et al., 1980). Not only do LC neuronal numbers decrease by ~25%, the level of NA in the brain also is reduced by 40% between the 4th and 9th decades of life (Marien et al., 2004), and this can be recapitulated in rodents, where the reduction is generally less prominent than in humans (Leslie et al., 1985).

LC neurons are sensitive to chronic inflammation and microglial activity, events that occur naturally during aging, yet are exacerbated in neurodegenerative diseases (Bardou et al., 2014). This is also the case with the effects of NA on microglia in LC target regions, where beta-amyloid clearance is attenuated when NA levels are lower in older subjects (Kong et al., 2010; Le et al., 2025). Therefore, in those treatments that target microglial phagocytosis, the level of NA is viewed as an important regulatory factor in that LC neurodegeneration could exacerbate AD progression (Heneka et al., 2010). NA itself is neuroprotective and anti-inflammatory by inducing expression of inhibitor kappa B-alpha (IκB-*α*) in neurons, and nitric oxide synthase type 2 (NOS2) in glial cells; while also reducing tumor necrosis factor alpha (TNF-α) expression in microglia and interferon gamma (Ifγ) in astrocytes following inflammation (Sara, 2009). Other key neuroprotective proteins are also negatively affected by the reduction in NA in target areas (Biagioni et al., 2024). Finally, capillary blood flow and water permeability are regulated by NA, and the same exacerbating effect of LC neurodegeneration can therefore be found in AD and PD compared to normal aging in terms of tight junction disruption at the blood–brain barrier (BBB) and blood volume / oxygen demand homeostasis (Mann, 1983; Kalinin et al., 2006; Bekar et al., 2012).

Age-related degeneration of the LC causes deficits in attention, episodic and working memory, and cognition (Mather, 2018; Hämmerer et al., 2018; Dahl et al., 2019), and quite possibly through the mechanisms described above. Since AD occurs in the context of aging, AD could be seen as one of a continuum of events that broadly fall under the category of aging. It is therefore important to identify what the key differences are between normal aging of the brain and AD.

### 2.2 Phosphorylation of tau protein

Tau protein phosphorylation is known as one of the primary causes of LC degeneration during aging and disease (Omoluabi et al., 2025; Satoh and Iijima, 2019). Tau proteins are a group of six highly soluble protein isoforms that play a role in maintaining the stability of microtubules in axons and are abundantly expressed in CNS neurons (Bachmann et al., 2021; Barbier et al., 2019). Tau proteins are normally phosphorylated to preserve microtubule function; however, under pathological conditions, these proteins become hyperphosphorylated, insoluble, and inefficient. Hyperphosphorylated tau exhibits a reduced affinity for microtubules, leading to destabilization of the axonal cytoskeleton and impaired intracellular transport of organelles and vesicles. This disruption hampers synaptic function and contributes to neuronal degeneration observed in aging and neurodegenerative diseases (Rodriguez-Martin et al., 2013). Recent studies have shown that hyperphosphorylated tau accumulates in neurons, causing neurite degeneration, loss of viable synapses, and behavioral abnormalities, even in the absence of fibrillar tau structures (Watamura et al., 2025). The hyperphosphorylated tau itself does not immediately cause neuronal damage, instead accumulating over decades in a soluble “pretangle” state prior to the first signs of aggregated neurofibrillary tangles, tauopathy or any clinical manifestations of mild cognitive impairment. This process occurs in the LC long before any other brain regions (Giorgi et al., 2019; Braak et al., 2011). In the later stages, the presence of amyloid exacerbates tauopathy and this in turn may mediate Aβ neurotoxicity in the cortex in humans and also in Aβ-overproducing transgenic rat model (Cohen et al., 2013). Interestingly, it has been shown that the accumulation of the phosphorylated tau protein co-localizes with active caspase enzymes in LC neurons. Since caspase enzymes are involved in programmed cell death, it is possible that the accumulation of the phosphorylated tau protein in the LC neurons plays a role in their degeneration (Wai et al., 2009). These findings suggest that the LC nucleus contributes to the onset and progression of AD (Bueicheku et al., 2024; Ehrenberg et al., 2017).

During the course of the progressing degeneration, compensatory mechanisms—such as high NA turnover (Hoogendijk et al., 1999) and LC hyperactivity (Weinshenker, 2018; Kelberman et al., 2023) in both humans and rodent models of AD—become increasingly dominant. This model of LC hyperactivity predicts that as establishment of hyperphosphorylated tau within the LC results in increased neuronal activity causing pre-MCI (“prodromal”) symptoms, such as anxiety, hyperarousal (Kelberman et al., 2023; Kelberman et al., 2022), and in humans, aggression (Bachman and Rabins, 2006; Ballard and Corbett, 2013; Scarmeas et al., 2007). As the disease progresses, phosphorylated tau proteins form neurofibrillary tangles (NFT) in the LC, intensifying cell loss, and MCI, eventually resulting in AD diagnosis (Orlando et al., 2023). Kelly et al. have shown that in AD, tau pathology of LC neurons decreased the expression of the genes related to mitochondrial function (Kelly et al., 2017). Experimentally, LC neuronal activity transitions from hyper- to hypo-activity in the later stages of AD (Kelberman et al., 2023), and chemogenetic activation of the LC reverses cognitive deficits, indicating a causative relationship (Rorabaugh et al., 2017). Combined, evidence from human and animal models indicates that tau pathology within the LC is a critical driver of Alzheimer’s disease onset and progression. Early hyperphosphorylation of tau alters LC neuronal activity, contributing to prodromal symptoms such as anxiety and hyperarousal. With disease advancement, tau aggregates into neurofibrillary tangles, accelerating LC cell loss and cognitive decline. These findings highlight the close interplay between tau accumulation and LC dysfunction in shaping the trajectory of Alzheimer’s disease.

### 2.3 High metabolic demand and oxidative stress in LC neurons

Metabolic demands are placed on LC neurons due to their involvement in regulating a significant number of basic physiological functions, including arousal, attention, stress responses, cardiovascular regulation, and sleep–wake cycles, which require sustained spontaneous activity in both normal and stressful conditions. As we explained in part 1–2, LC neurons can adjust their firing across a roughly tenfold range (from less than 1 Hz up to ~5 Hz) depending on behavioral state and external stimuli, allowing flexible modulation of arousal and attention. In stressful situations, firing rate increases or phasic bursts occur to facilitate adaptive behavioral responses. Pacemaker activity, due to the presence of L- and T-type voltage-gated calcium channels, permits maintenance of activity even after the interruption of glutamatergic and GABAergic inputs (Matschke et al., 2015). Consequent high levels of intracellular Ca^2+^ will, in turn, promote the mitochondrial oxidative stress of these neurons (Sanchez-Padilla et al., 2014). And high levels of oxidative stress make neurons susceptible to DNA damage (Wang and Michaelis, 2010), which is probably one of the causes of the selective vulnerability of LC neurons (Zhan et al., 2019). Also, the activation of the mitochondrial-dependent programmed cell death pathway and caspase 9 enzyme has been reported in the LC neurons of PD and Lewy body dementia (Kawamoto et al., 2014) as well as AD patients (Rohn et al., 2002).

Another metabolic demand on LC neurons is related to their morphology. LC neurons have elongated, diffuse and unmyelinated projections that extend throughout the CNS. Subsequently, these neurons require more metabolic support than neurons with shorter axon lengths and more sparse projections (Sara, 2009). Oxidative stress and neurotoxicity might be more consequential in the LC than in other areas, and therapeutic protection of LC neurons might even be an avenue to delay or mitigate AD onset.

### 2.4 Widespread proximity of LC neurons to CNS capillaries

Anatomical investigations have revealed that the blood supply of the LC is distinct from most other brain regions except the paraventricular and supraoptic nuclei (Samuels and Szabadi, 2008). The results of an old study conducted in 1940 in non-human primates revealed that the cell body of each LC neuron was surrounded by two or more capillaries (Finley and Cobb, 1940). Additionally, LC neurons send projections to the vast majority of CNS capillaries and the astrocytic end-feet forming the BBB. Considering the total number of LC neurons and the total length of the capillaries in the normal human brain, Pamphlett has hypothesized that each LC neuron innervates 20 m of capillaries on average (Pamphlett, 2014).

These special features enable the LC to maintain the BBB by releasing noradrenaline (NA), which acts on both endothelial cells and astrocytes to regulate barrier integrity, tight junction protein expression, and endothelial pinocytotic activity (Sobral et al., 2025; Verkhratsky et al., 2023). Additionally, NA released by LC neurons can selectively increase blood flow in the most active regions employed during vigilance, supporting higher metabolic demands during vigilance (Bekar et al., 2012). Lastly, additional vulnerabilities for the LC lie in its anatomical location, in the “corner” of the base of the fourth ventricle. This may lead to neuronal exposure to neurotoxins and inflammatory cytokines present in the cerebrospinal fluid stream (Mravec et al., 2014; Zorec et al., 2017).

Because LC neurons are constantly and intensively exposed to blood flow, they are more likely to be affected by potential toxins carried in blood, even at low concentrations, than other areas of the brain. This vulnerability has been hypothesized by Giorgi et al. (2020). Generally, two major groups of toxins can affect the LC nucleus. The first group consists of transfer toxicants that, after passing through the BBB using norepinephrine transporters (NET) located in the axon terminals of LC neurons, enter them. Then they reach the LC neurons’ soma by retrograde transport (Font et al., 1982; Gilsbach et al., 2006). The sensitivity of LC neurons to transfer toxicants depends on the presence of NET that is abundantly expressed in these neurons (Bonisch and Bruss, 2006). DSP4 is a selective neurotoxin for the LC-NA system in rodent and bird brains that selectively destroys LC neurons and is used in laboratory studies to lesion the LC. It was discovered in 1973 that DSP4 can easily cross the BBB. It selectively binds to NET, irreversibly inhibits it, enters the cell and nucleolus, and destroys the axon and cell body (Ross and Stenfors, 2015). It must be mentioned that there is substantial variation in the amount of damage caused to the LC axon terminal relative to the cell bodies in various species (Ross and Stenfors, 2015), though a major contributor to the observed lack of consistency may be due to experimental design (Szot et al., 2010). These differences are not confined to the LC-NA and are also found in the degree to which DSP4 affects the serotonergic system in various species. Yet the specificity to LC-NA over non-coerulear NA terminals persists (Fornai et al., 1996), suggesting a particular vulnerability of the LC-NA system even in species where it is not as pronounced. DSP4-like substances are found in cigarette smoke, car exhaust, and some plants. On the other hand, it has been found that toxins and environmental pollution can reduce LC neuronal NA reuptake (Pamphlett, 2014; Ross and Stenfors, 2015). The second group is intracellular toxins that pass through the BBB like the first group, but they use cotransmitters’ transporters in addition to NET for entering the axon terminals of LC neurons. Also, intracellular toxins can directly enter the cell body of LC neurons using NET in the membrane of the soma (Pamphlett, 2014). Heavy metals such as mercury, lead, silver, and bismuth belong to this group and accumulate inside the cytoplasm of the LC cells. Pamphlett et al. have detected LC neurons filled with environmental toxins and heavy metals with variable concentrations in the human brain (Pamphlett, 2014; Pamphlett and Kum Jew, 2014; Pamphlett et al., 2020), yet in human AD the population of LC neurons enriched in heavy metals were not the same as those enriched in hyperphosphorylated tau (Pamphlett and Kum Jew, 2015). The authors suggest a model by which the toxic metals interact with neuromelanin, discussed below, and the neurofibrillary tangles resulting from hyperphosphorylated tau, as discussed above, both interact to reduce NA transmission and BBB function.

### 2.5 Neuromelanin

Most LC neurons contain neuromelanin (NM), which is a by-product of catecholamine synthesis oxidation. NM can have both neuroprotective and neurotoxic effects. In natural conditions, NM can chelate environmental toxins, including heavy metals such as iron, cadmium, copper, and mercury, and reduce their toxic effects (Zecca et al., 2008; Zucca et al., 2017). NM also removes excess catecholamines inside the cells (Font et al., 1982). But the stress resulting from aging, disease or the accumulation of toxins prevents the chelating effect of NM and causes toxicity (Giorgi et al., 2020). In this way, since NM granules do not have an enclosed membrane, these accumulated toxins may be released in the cell cytoplasm (Doublea et al., 2008) and upon cell death are released into the extracellular space to propagate neurodegeneration to neighboring neurons (Zucca et al., 2017). Dopaminergic neurons in the substantia nigra also contain NM; however, in AD, the substantia nigra exhibits more resistance to neuronal loss than the LC, while the opposite is seen in PD, where greater neuronal loss in the substantia nigra is found than in the LC nucleus. Therefore, NM cannot be the only vulnerability factor, though it may interact with other factors leading to neurodegeneration in diseases such as AD (Iannitelli and Weinshenker, 2023).

The presence of NM in LC neurons is useful for identifying these cells after death and also *in vivo* imaging by NM-sensitive magnetic resonance imaging (NM-MRI), which can be correlated with cognitive function (Beardmore et al., 2021; Hämmerer et al., 2018). However, the MRI signal is not exclusively due to NM, as other factors such as lipids, proteins, water, etc. may also contribute.

### 2.6 Decreased expression of LC co-transmitters and trophic factors

Differences in expression of co-transmitters and receptors in the LC of AD patients have been hypothesized to be either neuroprotective or to expose these cells to vulnerabilities. For instance, though overall LC neurodegeneration was observed in one study, LC neurons co-expressing galanin were seemingly unaffected, as their numbers were roughly the same between AD and normal older human controls. Thus indicating a protective effect of galanin expression or signaling (Miller et al., 1999). On the other hand, a study by Adori and colleagues discovered a reduction of somatostatin receptor function in the LC nucleus of AD patients, which could be involved in the vulnerability of the LC (Ádori et al., 2015).

It has also been observed that the loss of afferent projections to the LC in AD has compounding effects on disease symptoms. For instance, lateral hypothalamic orexin B inputs significantly decreased with age in macaques, and this decrease was significantly correlated with the decrease in the expression of the tyrosine hydroxylase enzyme gene (Downs et al., 2007). This is even more pronounced in human AD (Oh et al., 2019). Indeed, a similar study in orexin (hypocretin)-deficient mice showed that chronic sleep fragmentation-induced Aβ_42_ accumulation was relatively reduced by the absence of orexin in the hippocampus, but this protective effect was not observed in the LC (Nick et al., 2022). The combined effect of orexinergic and LC neurodegeneration thereby contribute to the characteristic sleep deficiencies in AD (Parhizkar and Holtzman, 2025) which in turn worsen AD progression (Wang and Holtzman, 2020).

It has been determined that the growth and survival of LC neurons depends upon various neurotrophins that can be expressed in LC neurons or provided by retrograde transport from its target areas. The expression of these elements is controlled by NA (Remy et al., 2001). Therefore, changes in the function of the LC or NA levels can affect the expression of the trophic factors. Similarly, retrograde transport of trophic factors is critical for neuronal development and survival (Ito and Enomoto, 2016) and given the diversity of the LC targets, any pathological conditions in these structures might also contribute to LC neurodegeneration.

## 3 Clinical impact and prognostic value of LC vulnerabilities

According to the World Health Organization, AD and other dementias affect an estimated 55 million people (World Health Organization, n.d.) and somewhere between 69 and 315 million more people may be living with preclinical or prodromal symptoms (Gustavsson et al., 2023). This suggests that a staggering 384 million people may benefit from early diagnosis and prognosis of the disease and underscores the need for accurate, early diagnosis. In the US, Medicare pays for 80% of doctors’ visits, which can amount to around $26,000 per year for some of the newer AD treatments. While early diagnoses may incur more up-front costs (i.e., earlier treatment initiation), economic models suggest that later savings could be achieved by, for instance, contributing to a delay of patient institutionalization (Foldes et al., 2018; Jutkowitz et al., 2023; Weimer and Sager, 2009). Finally, given the similarities of the LC-NA system involvement in certain types of epileptic seizures (Giorgi et al., 2004) and PD (Gesi et al., 2000), advances in the field of AD could also inform clinical practice in a wide array of other diseases.

### 3.1 Correlation of Alzheimer’s Braak stages with LC dysfunction

Motivated by the observation that, though end-stage postmortem AD had very clear anatomical characteristics, the earlier stages did not, the husband-wife team of Heiko and Eva Braak described a series of morphological stages throughout AD progression in 1991 (Braak and Braak, 1991). These stages are a semiquantitative measure of the severity of tau-based neurofibrillary tangle (NFT), which is the main neuropathological hallmark of AD. They describe the NFT pathology from least to greatest within the medial temporal lobe memory circuit in AD using the Bielschowsky silver stain to visualize NFTs in the frontal, temporal, parietal, and entorhinal cortex, as well as the hippocampus. The Braak stages were based upon the distribution and severity of NFT pathology: Stages I and II indicate NFTs mainly confined to the entorhinal region. Stages III and IV point towards the involvement of limbic regions such as the hippocampus. Stages V and VI signify moderate to severe involvement of the neocortex (Braak and Braak, 1991). According to the Braak stages, LC is the first brain area to show specific alterations, many years before the clinical onset of AD (Braak et al., 2011), involving “pretangle” tau as discussed above. Cassidy et al. investigated the association between LC MRI signals and AD patients’ Braak stage, which was extrapolated from tau PET scans. The findings show the direct relation between LC integrity and Braak stages (Cassidy et al., 2022). Consistent with this, stereological analyses of human postmortem brainstems were performed to estimate LC volume and neuronal population in healthy and AD patients. The results revealed that as the Braak stage increases by 1 unit, the LC volume decreases by 8.4% (Theofilas et al., 2017) and the decline in neural density correlates with cognitive decline (Wilson et al., 2013). Further explorations of AD have disclosed that the accumulation of hyperphosphorylated tau in LC neurons leads to their degeneration as the Braak stages progress (Van Egroo et al., 2023; Jacobs et al., 2022). These are potential implications of LC pathology as an early-stage biomarker for the diagnosis of AD.

### 3.2 LC degeneration and memory deficits in Alzheimer’s disease

The Braaks were initially hesitant to correlate their anatomical findings with disease symptomatology given the high inter-individual variability, particularly in early stages of the disease. However, the functional connections of the LC with several brain structures involved in learning and memory, including the cortical regions and hippocampus, make this line of inquiry stand out. The decline in noradrenergic transmission is consistent with the cognitive impairment seen in AD. Apart from NA, LC neurons also produce dopamine, and they can corelease both catecholamines to influence synaptic plasticity and memory in the hippocampus (Ciampa et al., 2022). *In vivo* research on the integrity of the LC consistently shows an association with memory deficits (Jacobs et al., 2022; Dahl et al., 2022). Dahl et al. have recently put forth the hypothesis, based on rodent (Dahl et al., 2023b) and human (Dahl et al., 2023a) studies, that the declining catecholaminergic input to the hippocampus in both normal and AD aging leads to decreased synaptic plasticity and subsequent decline in memory performance.

### 3.3 The need for earlier Alzheimer’s diagnoses

A new class of AD medications, monoclonal antibodies directed against Aβ, has shown limited delay of disease progression in ~30% of patients (Boxer and Sperling, 2023). This may be due to the clinical trial inclusion criteria: participants needed to show clinical symptoms and signs of deteriorating cognitive performance. Since accelerated Aβ deposition and initial neurodegeneration is likely to have begun up to 10 years prior to symptom onset, preventive interventions rather than mitigation of disease progression has been proposed (Jucker and Walker, 2023). Longitudinal studies in preclinical / asymptomatic subjects, such as the AHEAD 3–45, are already underway (Rafii et al., 2023). Thus, the need for early AD diagnosis was also highlighted in a US Food and Drug Administration guidance for industry proposal (Food and Drug Administration, n.d.).

Prognoses and interventions beginning long before the onset of symptoms could also be directed at processes influenced by the LC such as the sleep/wake cycle. Sleep EEG is a potential biomarker of the relative “brain age” of individuals with the same chronological age (Sun et al., 2019). Chronic sleep disruption is a risk factor for AD (Parhizkar and Holtzman, 2025; Wang and Holtzman, 2020; Bubu et al., 2017; Himali et al., 2023; Neylan and Walsh, 2024), and prolonged sleep durations may protect cognitive function in the prodromal stage of the disease in some prospective AD patients (Baril et al., 2024), which is a high-priority area of research (Andre et al., 2025). Possibly, a combination of AD biomarkers in conjunction with circadian and sleep measures could even assess individuals’ probability of progressing to AD (Yang et al., 2024). In light of this, it is interesting to speculate whether the sleep disruption could be caused by prodromal LC hyperactivity (Mayer et al., 2024). Nocturnal awakenings are associated with decreases in LC integrity as measured by MRI in AD patients (Van Egroo et al., 2021), suggesting that longitudinal studies of LC integrity, sleep/wake alterations prior to AD symptom onset may offer early prognostic value (Van Egroo et al., 2022). In later stages, so-called “sundowning,” occurs in ~20–45% of people with AD and has been linked to LC neurodegeneration (Yang et al., 2024; Garcia-Rill, 2019; Oh et al., 2022), and hyperactivity (Fitzgerald, 2021). Counteracting sleep loss itself could even one day be used to delay disease onset. The consensus view is that while Aβ and tau accumulate in the interstitial fluid during wakefulness, they are cleared during the deepest stage of non-REM sleep, slow-wave sleep (Holth et al., 2019), and that this precedes cognitive decline and is related to autonomic regulation (Chen et al., 2023). The neuroprotective role of non-REM sleep in AD pathology has also been confirmed in humans in both correlational (Zavecz et al., 2023) and pharmacological (Lucey et al., 2023; Zhou and Tang, 2022) studies, further opening up the possibility of shunting the deleterious effects of LC overactivation on Aβ removal during slow-wave sleep (La et al., 2019; Ashford, 2019), prior to symptom onset.

While currently available blood biomarkers provide fast, inexpensive and accurate test results for those with MCI (Li et al., 2022; Brand et al., 2022; Ashton et al., 2024; Barthelemy et al., 2024), the sensitivity and specificity of these tests are often considerably lower in presymptomatic individuals. On the other hand, there has been tremendous progress recently in advancing LC imaging *in vivo* in humans as a way to diagnose neurodegeneration and earlier AD prognosis (Kelberman et al., 2020). The potential for the longitudinal prediction of future cognitive abilities based on catecholaminergic size in MRI has recently been demonstrated (Dahl et al., 2023a) and thus posits that the LC is not only a target of AD symptom intervention but also an early diagnostic/prognostic center for the development of AD (Dahl et al., 2023b). In the following section, we review LC imaging in relation to AD diagnosis.

### 3.4 Can LC imaging improve Alzheimer’s diagnoses?

Structural MRI scans with higher resolution are minimally invasive and widely available compared to other techniques such as PET, so appear to be an ideal method to study the LC. Aggregation of hyperphosphorylated tau protein results in reduced LC integrity in the neurodegenerative disease state (Jacobs et al., 2022; Dahl et al., 2022). Betts and colleagues showed that lower LC contrast, in the rostral and middle thirds of the LC, is correlated with CSF Aβ levels (Betts et al., 2019; Betts et al., 2019). These findings are in agreement with the Dahl et al. study highlighting the importance of rostral parts of the LC in memory performance (Dahl et al., 2019). Structural covariance of the LC with the whole brain is also correlated to subjective and objective memory loss (Fu et al., 2021; Tang et al., 2023). Together, this suggests that LC contrast studies could be used as a diagnostic tool to assess the AD onset and progression.

It should be noted that LC imaging parameters are also negatively correlated with normal aging (Betts et al., 2017) and aging-related behavioral changes (Liu et al., 2019; Liu et al., 2020) as well as brain correlates of cognition in later life (Bachman et al., 2021). Though when comparing to subjects who later display cognitive decline, no differences are noted in the cohort of cognitively intact individuals (Giorgi et al., 2022). Nevertheless, the specific alterations seen in AD patients’ LC imaging correlate well with posthumous LC cell counts (Theofilas et al., 2017; Betts et al., 2019; Keren et al., 2015).

Diffusion tensor imaging provides a detailed, microstructural view of the brain by quantifying the diffusion of water and other fluids, allowing imaging of axon fibers (Basser et al., 1994). Using DTI we can obtain more information about LC axonal dysfunctions such as myelination, integrity or size of wide and diffuse projections of the LC to its target regions (Kelberman et al., 2020; Lenglet, 2015). Moreover, DTI can reliably detect differences in the integrity of the LC projections related to resilience to sleep deprivation (Quan et al., 2024) and ageing (Svendsen et al., 1986). Recently, a reduction in LC integrity was found in older individuals, and this was correlated with a decline in memory performance (Langley et al., 2020). And a reduction in fiber integrity of the LC cortical projections was found in AD patients (Sun et al., 2020), which is likely due to LC cell loss rather than fiber denervation (Lin et al., 2024; Quattrini et al., 2024). As advances in resolution continue to be made, for instance via MR relaxometry (Bae et al., 2025), early microstructural changes in the LC are becoming a reality.

Functional MRI (fMRI) displays regional and temporal changes in brain activity through changes in blood flow, though it is complicated for a variety of reasons. First, since LC neurons fire using two separate patterns, tonic and phasic, each may recruit specific functional connectivities. In addition, these neurons do not always fire synchronously. Therefore, attention should be paid to technical analysis considerations when using LC fMRI (Kelberman et al., 2020). A recent advance in imaging resolution and data analysis first developed using structural MRI (Hämmerer et al., 2018) and then fMRI (Ludwig et al., 2024), with the former study showing a correlation between older age LC integrity and salient memories, and the latter study showing age-related increases in LC activity during learning tasks. Another recent study has determined “LC-to-pons” parameters that reliably distinguish between AD and age-matched controls (Dordevic et al., 2017), and this “T1-weighted turbo spin echo” scan approach (Takahashi et al., 2015) has been shown to be longitudinally reliable as well (Tona et al., 2017). Beyond that, and contrary to the idea that LC-NA connection with the striatum is weak, resting-state fMRI has revealed that LC connectivity with the salience networks in older people declines (Lee et al., 2020; Liebe et al., 2020). Functional MRI studies have revealed that a stronger connection between LC and the parahippocampal gyrus is associated with better memory performance (Sara, 2009; Jacobs et al., 2015). Finally, ultra-high resolution 7 T fMRI has shown much promise in overcoming the size limitations of LC imaging. Studies of LC functional connectivity (FC) in ageing have shown linear decreases in FC between the LC and some areas, whereas non-linear relationships exist with others (Jacobs et al., 2018), an association of emotional memory and emotion regulation along the rostro-caudal extent of the LC (Vereb et al., 2023), and a slower memory decline in individuals ageing with higher memory capacity (Mijalkov et al., 2025).

Positron Emission Tomography (PET) visualizes metabolic and other physiological activity using radiotracers. PET is associated with greater costs and lower resolution compared to structural MRI, but its advantage is that it can be used to measure LC dysfunction in the terminal regions. Norepinephrine transporters, which are expressed in all parts of LC cells, can be assessed with PET in regions innervated by the LC to evaluate the LC fiber integrity (Arakawa et al., 2008; Brumberg et al., 2019; Chen et al., 2020; Ding et al., 2006; Moriguchi et al., 2017). Significant interactions of tau tracers with neuromelanin make direct PET tau imaging of the LC currently unadvisable (Betts et al., 2019; Krohn et al., 2023; Marquie et al., 2015). Also, using the metabolic activity of the LC as measured via [^18^F]fluorodeoxyglucose (FDG) as a biomarker for Alzheimer’s is still somewhat uncertain in that one study found no correlation (Liu et al., 2021), though another found increased activity in the preclinical stages correlated with attenuated memory decline (Koops et al., 2025). Importantly, however, lower LC integrity as measured by LC-MRI is correlated with greater cortical tau pathology as measured by PET, as well A-*β* and cognitive measures (Jacobs et al., 2022), and lower regional cortical glucose metabolism as measured by [^18^F]FDG in individuals with dementia and MCI (Aghakhanyan et al., 2023), and this is correlated with cognitive performance in patients with AD (Khosravi et al., 2019).

## 4 Conclusion

The unique neuronal architecture and morphology, physiology, activity and copious ascending and descending projections allow the LC to be involved in a wide variety of functions such as sleep and wakefulness, the stress response, attention, memory and sensory processing, etc. This also exposes the LC to vulnerabilities leading to neurodegeneration and propagation of neurodegenerative species to other brain areas. It is clear that the LC is one of the earliest affected areas in Alzheimer’s disease, and contemporary imaging techniques are now able to detect these foundational changes. These insights will be leveraged into expanded fundamental knowledge of AD initiation and progression, and further refinement should lead to earlier disease detection and/or treatment and lifestyle modification options.

Many factors can play a role in the damage of LC neurons, which show a decline during aging that can be correlated to the increased activity of microglia, the accumulation of phosphorylated tau protein, the accumulation of environmental toxins, and the reduction of trophic factors from the target areas of the LC. Therefore, additional damage to this nucleus during disease progression can be the result of the aggravation of these natural processes. This exacerbation may be the result of pathological and inflammatory events in the target areas of the LC nucleus, which reduce the production of trophic factors and sometimes produce toxic substances. On the other hand, toxins act selectively on LC neurons. LC damage has also been observed in many laboratory models of neurodegenerative diseases and large-scale animal studies are being conducted to understand the dimensions of these effects. In medicine, translational brain imaging studies are a big step in this field.

There are some caveats to note. Brain imaging is a very expensive and somewhat intrusive endeavor. While there is some hope that over time, techniques will be refined and standardized in a way that will lower costs and enhance access (Engels-Dominguez et al., 2024), the high fixed costs of imaging will likely not make any of these techniques routine outside of research settings anytime soon, if ever. Nonetheless, the demand for early testing—decades prior to symptom onset—amongst the general population is exceedingly high according to a recent Alzheimer’s Association survey (Alzheimer's Association Report, 2025). Imaging of the LC, with the early signs of AD reviewed here, can assist in this. Most of the studies cited in this review article state that longitudinal studies, and the combination of structural and functional imaging are needed to understand the progression of LC damage in AD better, sentiments echoed in a prior review article (Engels-Dominguez et al., 2023). If costs could be reduced and resolution increased with future advanced technologies, criteria could be developed to triage subjects for imaging in a wider population.
